# Monkeypox: A Comprehensive Review of Transmission, Pathogenesis, and Manifestation

**DOI:** 10.7759/cureus.26531

**Published:** 2022-07-03

**Authors:** Jasndeep Kaler, Azhar Hussain, Gina Flores, Shehreen Kheiri, Dara Desrosiers

**Affiliations:** 1 Medicine, Xavier University School of Medicine, Oranjestad, ABW; 2 Healthcare Administration, Franklin University, Columbus, USA; 3 Medicine, Rutgers University, New York, USA; 4 Pharmacology, Touro College of Pharmacy, New York, USA; 5 Cardiology, St. Joseph’s College, New York, USA

**Keywords:** poxviridae, chordopoxvirinae, poxvirus, orthopoxvirus, monkeypox

## Abstract

As the fear of the coronavirus disease 2019 (COVID-19) pandemic subsides, countries around the globe are now dealing with a fear of the epidemic surrounding the prevalence of monkeypox cases in various regions. Previously endemic to regions of Africa, the majority of monkeypox cases associated with the 2022 outbreak are being noted in countries around Europe and in the western hemisphere. While contact-tracing projects are being conducted by various organizations, it is unknown how this outbreak began. Monkeypox virus is one of the many zoonotic viruses that belong to the Orthopoxvirus genus of the Poxviridae family. Monkeypox cases received global attention during the 1970s, after the global eradication of smallpox. The smallpox vaccine provided cross-immunity to the monkeypox virus. Upon the cessation of smallpox vaccine administration, monkeypox cases became more prevalent. It was not until the 2003 US outbreak that monkeypox truly gained global attention. Despite the virus being named monkeypox, monkeys are not the origin of the virus. Several rodents and small mammals have been attributed as the source of the virus; however, it is unknown what the true origin of monkeypox is. The name monkeypox is due to the viral infection being first witnessed in macaque monkeys. Though human-to-human transmission of monkeypox is very rare, it is commonly attributed to respiratory droplets or direct contact with mucocutaneous lesions of an infected individual. Currently, there is no treatment allocated for infected individuals, however, supportive treatments can be administered to provide symptom relief to individuals; Medications such as tecovirimat may be administered in very severe cases. These treatments are subjective, as there are no exact guidelines for symptom relief.

## Introduction and background

With the first human case reported in 1970 in a nine-month-old boy from the Democratic Republic of Congo, monkeypox is once again causing reason for panic as outbreaks are occurring across the western hemisphere. Endemic to central and western Africa, human monkeypox is a rare viral zoonosis that has been associated with the 2003-04 outbreak in the United States (US) [1].

**Figure 1:**
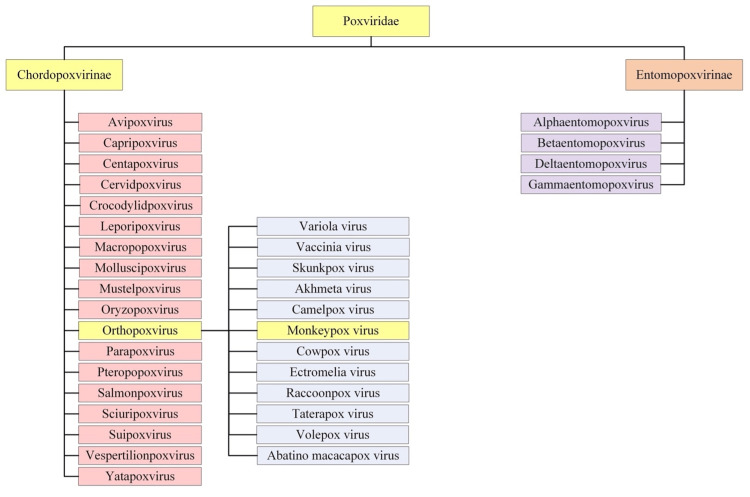
Taxonomy and Classification of Monkeypox within Poxviridae Lineage

Monkeypox is one of the many zoonotic viruses that belong to the Orthopoxvirus genus of the Poxviridae family, as presented in Figure 1. Isolated from various animals the Poxviridae viruses are large, enveloped, double-stranded DNA viruses [2]. The major hosts of Poxviruses are rodents, rabbits, and non-human primates, which can occasionally be transmitted to humans facilitating the occurrence of human-to-human transmission [3]. Taxonomically, the Poxviridae family is further categorized into two families: Entomopoxvirinae and Chorodopoxvirinae. The subfamily classification is based on whether the virus will infect insects, such as Entemopovirinae, or infect vertebras, as is the case with Chorodopoxvirinae [4]. The Chorodopoxvirinae family is further classified into 18 genera, as depicted in Figure 1. Each of the 18 genera within the Chorodopoxvirinae subfamily list several viruses, the majority of which are of zoonotic origin.

The clinical presentation of the monkeypox virus is similar to that of smallpox [5]. Monkeypox is less fatal than smallpox with case fatality rates of approximately 10% [6]. Prior to 1970, monkeypox was recognized only in non-human hosts(s) [7]. Monkeypox is described as enveloped, slightly pleomorphic, with a dumbbell-shaped core with lateral bodies [8]. There are two distinct genetic clades of the monkeypox virus: the central African (Congo Basin) clade and the West African clade [9]. Of the two genetic clades, historically, the Congo Basin clade has caused greater severity in disease and is thought to be more virulent [9-10]. A greater degree of morbidity, mortality, human-to-human transmission, and viremia was associated with the Congo Basin clade of human monkeypox disease, visible during the 2003 US outbreak [7,10].

Genome comparisons of the west and central African strains yielded a set of candidate genes that may be involved in the differentiating clade virulence [10]. The open reading frames in the west African clade contained deletions and fragmentations that contribute to its reduced virulence [10-11]. Central African monkeypox prevents T-cell receptor-mediated T-cell activation, prohibiting inflammatory cytokine production in human cells derived from previously infected monkeypox patients [10]. Hammarlund et al. observed that T-cell mediated cytokine responses were decreased by 80% in the presence of a low viral load of monkeypox, suggesting that monkeypox may produce a modulator that suppresses host T-cell responses [12]. The monkeypox virus inhibitor of complement enzymes (a gene that inhibits complement enzymes) is absent in west African strains, and it has been implicated as an important immune-modulating factor contributing to the increased virulence of central African strains [10,13-14]. Moreover, the central African strain selectively downregulates the host responses, specifically, apoptosis in the host [10]. Transcriptional studies have shown that central African monkeypox appears to selectively silence the transcription of genes involved in host immunity during infection [6,10].

Poxviruses are large, linear, double-stranded DNA viruses with a genome size ranging from 130 to 360 kbp that replicate in the cytoplasm of vertebrate or invertebrate cells [15-16]. DNA viruses typically replicate and express their genomes in the nucleus, making extensive use of cellular proteins, however, this is not the case for poxviruses [16]. Poxviruses are different in the sense that they rely heavily on virus-encoded proteins that enable them to replicate in the cytoplasm [17]. The central part of the genome contains genes involved in key functions, such as transcription and virus assembly, whereas those located at the termini are involved in virus-host interactions [15,18]. Of more than 150 genes encoded by poxviruses, 49 are common to all sequenced members of this family and 90 are common within the subfamily of chordopoxviruses [19]. The majority of these conserved genes among the viruses are related to viral function and form the central part of the genome [15].

Due to the larger size of poxviruses, it makes it harder for viruses such as monkeypox to breach host defenses by passing through gap junctions. The larger size of the virus also makes it difficult for the virus to replicate rapidly and orthopoxviruses need a more comprehensive strategy to survive within the host [20]. The larger size of the orthopoxviruses alerts the immune system of the individual very early on and thus, generates an immune response very easily [20-21]. To be able to evade the host immune system, orthopoxviruses are equipped with a set of molecules encoded by virulence genes that will act as modulators by being directed against components of the host’s immune system [20]. These proteins that are responsible for modulatory actions against the host’s immune response can be categorized into two groups according to whether they worked intracellularly or extracellularly.

**Figure 2:**
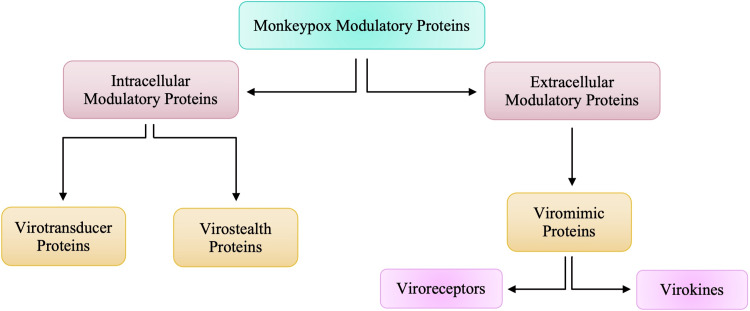
Intracellular and Extracellular Modulatory Proteins of Monkeypox

These proteins that are responsible for modulatory actions against the host’s immune response can be categorized into two groups, as highlighted in Figure 2. Intracellular proteins are virotransducer proteins and virostealth proteins. The virotransducer proteins act by playing a role in interfering with the cell’s ability to respond to the infection, including the oxidative burst and apoptotic pathways [20-21]. The virostealth proteins, which also act intracellularly, reduce the likelihood of detection of the virus by the host’s immune system via the downregulation of immune recognition molecules such as the major histocompatibility complex class 1 (MHC 1) and CD+4 [15,20-21]. While there are two different types of intracellular modulatory proteins that aid monkeypox in evading the host’s immune response, there is only one type of extracellular protein, viromimic proteins. Figure 2 shows that there are two different classifications of viromimic proteins, and both function to modulate the immune system’s response. The viroreceptors are secreted or are present as cell surface glycoproteins that bind host cytokines and chemokines competitively and, thus, interfere with their actions [20-21]. Consequently, virokines form viral mimics of host cytokines, chemokines, and growth factors that are effective in both subverting host responses that are detrimental to virus survival and in promoting responses appropriate for viral replication and spread [5,20]. These modulatory proteins work simultaneously to evade the host’s immune system to allow for viral replication. Without the presence of these proteins, Orthopoxviruses such as monkeypox would be unable to evade the immune system.

Poxviruses’ replication cycle provides an insight into how the replication cycle of the monkeypox virus functions. As with other viruses, poxviruses also have proteins that enable and aid the binding of the virus to a cell, membrane fusion, and entry into the host cell. In the case of poxvirus, the mature virion (MV), which has a single membrane, and the extracellular enveloped virion (EV), which has an additional outer membrane, are disrupted before the fusion [16]. There are four viral proteins that are associated with the MV and all these collectively will facilitate the attachment of MV to a host cell by binding glycosaminoglycans or laminin on the cell surface [16-17]. Regardless of whether the MV or EV mediate infection, the fusion of the virus to the host cell is dependent on 11 to 12 non-glycosylated, transmembrane proteins that range in size from 4 to 43 kDa [16]. MVs are very stable and are thought to mediate transmission between host animals, whereas EVs have a fragile outer membrane and are specifically specialized for exiting the intact cell and spreading within the host [16,22].

Poxvirus DNA replication progresses within cytoplasmic structures that originally were called Guarnieri bodies and are now commonly called factories [23]. Each factory derives from a single infecting particle and in the early stages of infection, they are compact DNA-containing structures that are surrounded by membranes that seemed to drive from the cell’s rough endoplasmic reticulum (RER) [22-23]. These factories will enlarge with continuing DNA synthesis and will gradually adopt a more irregular appearance as cavities form containing viral mRNA and host translation factors [23-24]. In the later stages of the replication cycle, a complex of late gene products and a collective of viral membrane assembly proteins will act to dismantle the surrounding endoplasmic reticulum membranes and produce crescent-shaped structures as substrates for the assembly of the immature virions (IV) [24]. The IV is then processed into MV, which are the most abundant infectious species [23]. These MV will exit the cell via fusion with the cytoplasmic membrane.

Epidemiology

Monkeypox has presumably occurred in sub-Saharan Africa for thousands of years, ever since humans acquired the virus through direct contact with infected animals [25]. Monkeypox was not recognized as a distinct disease until 1970 when the eradication of smallpox revealed the continued occurrence of smallpox-like illness in rural areas [8,25]. In 1958, the monkeypox virus was first identified in laboratory monkeys at State Serum Institutes in Copenhagen, Denmark, and Africa for research purposes [8,21]. Monkeypox has become a disease of global public health importance after 2003 due to the first outbreak in the USA linked to infected pet prairie dogs [9]. Native prairie dogs housed with rodents imported from Ghana in Western Africa were thought to be the primary source of the outbreak as most of the infected individuals became sick after contact with pet prairie dogs [25-26]. In the summer of 2003, monkeypox had been identified to be the cause of a cluster of cases in the US Midwest [25]. Since 2003, several cases of monkeypox have been reported in various countries with the largest outbreak experienced in Nigeria in 2017 [8-9].

In an epidemiological modeling study, the authors reported the R0 value of monkeypox to be between 1.10 and 2.40 in countries where exposure to Orthopoxvirus species is negligible, R0 is referred to as the reproductive ratio, or in other words, the degree of transmissibility of the disease [27]. This value suggests that an epidemic of monkeypox is imminent in scenarios of imported human or animal cases [20,28]. The reported R0, as mentioned previously, suggests that each infected individual possesses the ability to infect one to two other people. Due to the transmissibility of the virus, it becomes imperative that an infected individual takes special measures to social distance and quarantine him/herself.

**Figure 3:**
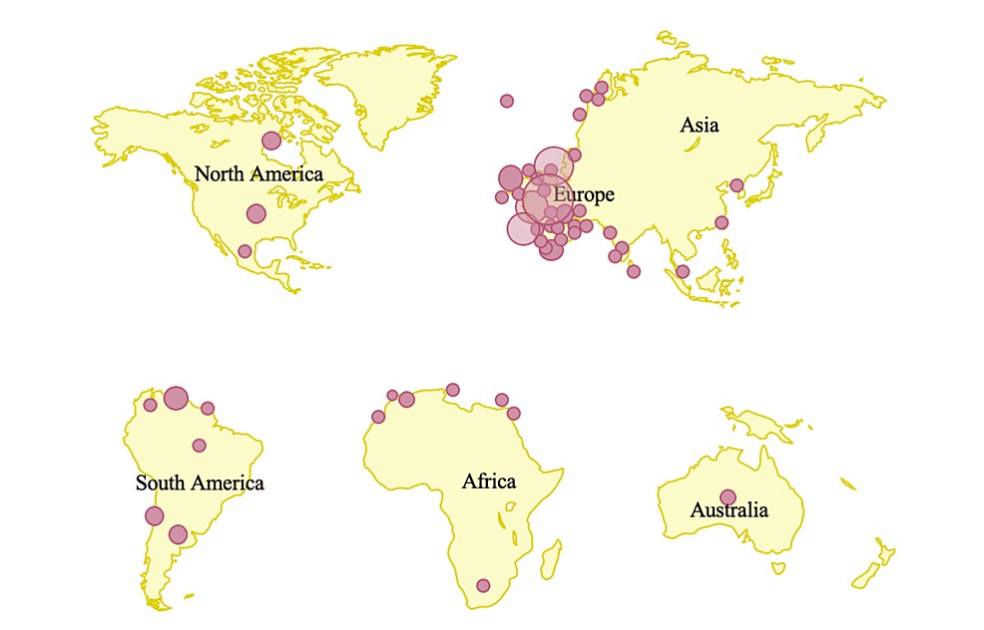
Current Regional Distribution of Confirmed Cases of Monkeypox

As of July 1, 2022, the Center for Disease Control and Prevention (CDC) confirms 5783 cases of monkeypox, distributed in 52 different countries around the globe [29]. Figure 3 is a visual depiction of the regional distribution of the cases globally. Many of the cases of monkeypox, currently, are concentrated within regions of Europe and within the western hemisphere [9]. Within Europe, current reports show that the largest number of cases are present in the United Kingdom. Currently, many of the confirmed cases of monkeypox are prevalent amongst individuals under the age of 40 years with a median age of 31 years [21]. This is a population born only after the discontinuation of the smallpox vaccination campaign, therefore further reflecting the lack of cross-protective immunity [20-21]. There is also a higher prevalence of monkeypox cases among males, however, the exact explanation for this is unknown.

## Review

Various orthopoxviruses share similar genetic and antigenic features and, as such, infection by any of these viruses may provide substantial protection against infection by the other viruses from the orthopoxvirus genus. Due to cross-immunity, an immune response against any illness caused by any orthopoxvirus will decrease the likelihood of an infection by a different orthopoxvirus. Historically, vaccination against smallpox had been shown to be protective against monkeypox [9]. Since the eradication of smallpox in the 1970s, the smallpox vaccine was also discontinued. The cross-immunity the smallpox vaccine provided against other orthopoxviruses has started to wane [21]. Those less than 50 years of age are less likely to have been vaccinated against smallpox and are, therefore, more likely and susceptible to becoming infected by other orthopoxviruses such as monkeypox. Widespread smallpox vaccination in central Africa during the global eradication campaign presumably caused a temporary reduction in the incidence of human monkeypox. The absence of immunity in the generation born since that following year and onward along with the increased dependence on hunting for food in areas devastated by the civil war has resulted in the re-emergence of the disease [25].

Even though monkeypox was first identified in Denmark in a macaque colony, the exact animal reservoirs, and initial animal-to-human transmission of the virus remain unknown [30-31]. It has been shown that in intermediate hosts, the virus can be transmitted from one animal to another and subsequently to humans [30]. The transmission of monkeypox from intermediate hosts to humans is what preceded the 2003 US Midwest outbreak, in which pet prairie dogs were thought to have contracted the virus from infected rodents that were being shipped to the US from Ghana [31-32]. Though the exact host reservoir for monkeypox is still unknown, there are data that suggest that monkeys are, like humans, incidental hosts and that the reservoir is likely to be one or numerous species of rodents or squirrels that inhabit the secondary forest of central Africa [25].

**Figure 4:**
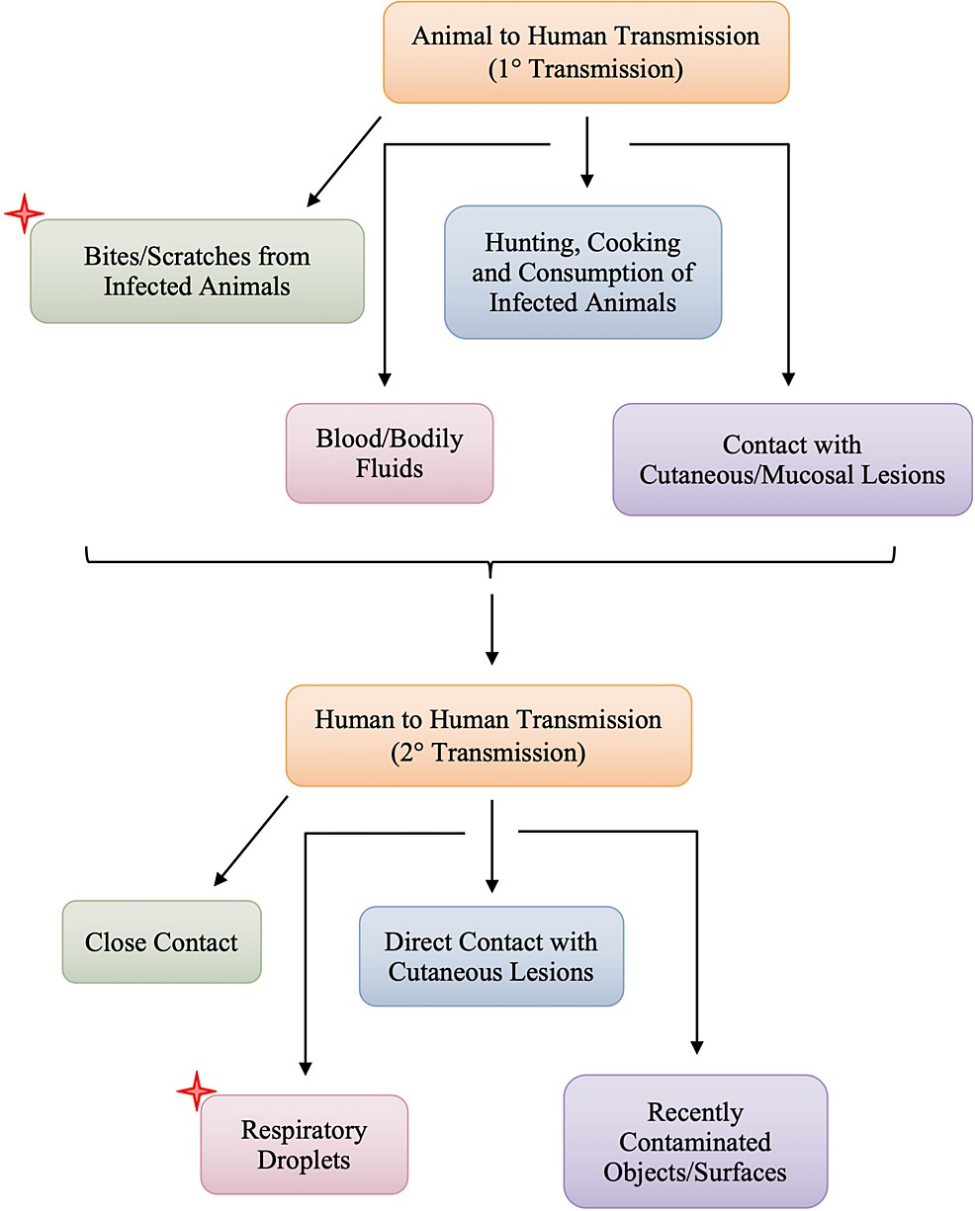
Suspected Modes of Transmission of Monkeypox to Humans

Monkeypox virus is believed to have several modes of transmission, all of which are associated with direct contact with infected animals or the with infected humans, as depicted in Figure 4. Human infections have been linked to contact with animals, but the precise exposure of a human case can be difficult to pinpoint in areas where contact with animals via household rodent infestations and the hunting or preparation of bushmeat from a variety of species is common [10]. The exact mode of transmission of monkeypox is still under investigation, however, the suspected modes of the transmission shown in Figure 4 are those that were listed as risk factors for monkeypox contraction by Bunge et al. [33]. Animal to human transmission is direct contact or exposure with infected animals and most commonly, due to bodily fluids such as saliva, respiratory excretions, or could be the exudate from cutaneous or mucosal lesions. Viral shedding via feces may represent another exposure source [34]. Exposure to feces of infected animals can be an important risk factor in endemic regions of Africa where resources and infrastructure are scarce, causing individuals to sleep outside, on the ground, or live near or visit the forest where infected animals are much more prevalent [33]. In areas of scarce resources, such as food, households are left with no choice but to hunt and cook small mammals, increasing their risk of exposure to monkeypox. Although human-to-human transmission is less common than animal-to-human, it usually involves respiratory droplets with prolonged face-to-face contact or contact with lesions of an infected individual [31]. Contaminated objects/surfaces, such as sleeping on the same bedding, living in the same household, or eating or drinking from the same dishes as an infected individual, are deemed a risk factor for viral transmission among individuals of the same household. Amid the current, ongoing monkeypox epidemic, it has also been observed that the disease is more common in males who have sex with males [20]. According to the World Health Organization (WHO), it is not yet known whether monkeypox is sexually transmitted or not, however, the transmission can be attributed to close contact [9,20]. The pathogenesis and pathophysiology of monkeypox begin from the transmission of the virus, whether it be human-to-human transmission or animal-to-human transmission.

**Figure 5:**
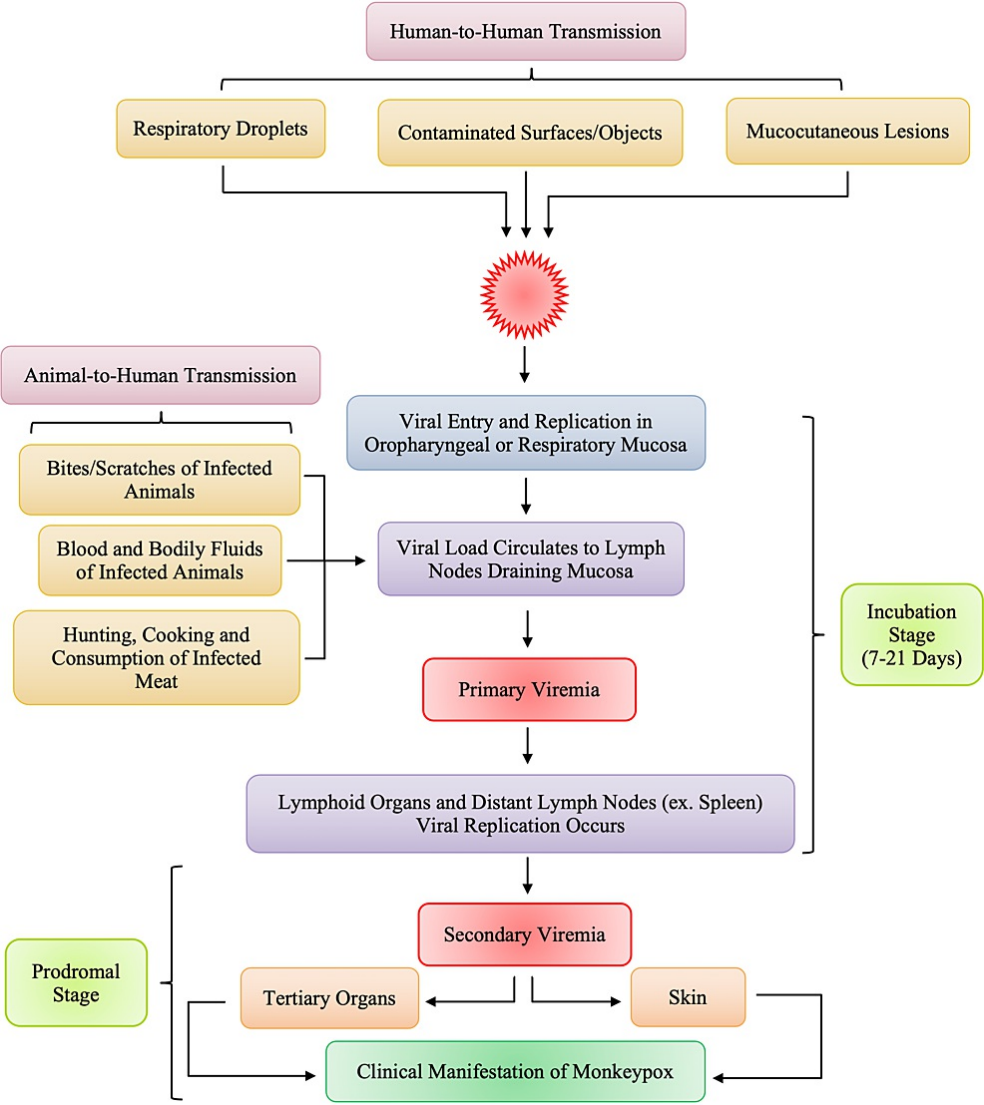
Proposed Pathogenesis of Monkeypox

The most common cause of human-to-human transmission, as rare as it is considered, is respiratory droplets. Figure 5 lists direct contact with contaminated objects/surfaces and direct contact with mucocutaneous lesions of an infected individual. Monkeypox virus follows a similar infectious pathway to smallpox, beginning with the exposure of the oropharyngeal or respiratory mucosa of the host. Following the viral entry, the monkeypox virus replicates at the site of inoculation; in human-to-human transmission, the site of inoculation is the respiratory and oropharyngeal mucosa. Following viral replication, in primary viremia, the viral load spreads to the local lymph nodes. In secondary viremia, the viral load will reach the distant lymph nodes and organs through circulation. The entire process represents the incubation period, typically lasting seven to 14 days with an upper limit of 21 days [35]. Clinical manifestation of monkeypox is not visible during the incubation stage and, therefore, the incubation period is not contagious. The symptoms and clinical manifestation of monkeypox can be correlated to the prodromal stage. During the prodromal stage, secondary viremia occurs from the lymphoid organs to the skin and tertiary organs such as the lungs, eyes, gastrointestinal tract, etc. It is during the prodromal state that an individual is deemed to be the most infectious. This is largely due to the presence of symptoms such as mucocutaneous lesions, and lymphadenopathy, among other non-specific symptoms.

**Figure 6:**
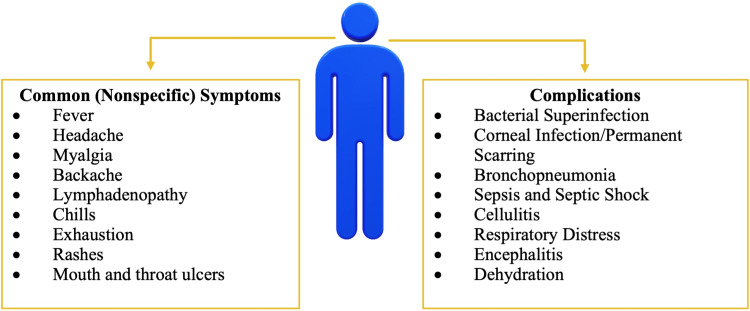
Categorization of Nonspecific Symptoms and Complications of Monkeypox

The common, nonspecific symptoms, as depicted in Figure 6, begin to develop one to two weeks after an individual has been infected with the monkeypox virus [36]. During the prodromal stage, nonspecific symptoms triggering the immune system emerge such as fever, lymphadenopathy, myalgias, etc. Due to the nonspecific nature of these initial symptoms, an infected individual may attribute these symptoms to seasonal flu or the common cold. Initial activation of the immune system will always cause enlargement of lymph nodes, including maxillary, cervical and inguinal, occurring in synchrony with the onset of fever [20]. In cases prior to the 2022 outbreak, rashes would be observed appearing one to three days after the onset of fever and lymphadenopathy. In the emergence of these new cases, Harris states that for some patients, those prodromal symptoms might be mild, or not even noticed at all, suggesting that some individuals may not be aware of any symptomology at all, until the appearance of the rash [35]. In typical cases, the fever often declines on the day of, or up to three days after, the onset of the rash [10]. The rash will first appear on the face and will quickly appear in a centrifugal distribution across the body [10,20]. A centrifugal distribution means that there will be more lesions on the extremities and the face rather than on the abdomen and trunk. Figure 6 mentions lesions that are often noted in the oral cavity, and these lesions cause difficulties with eating and drinking and thus, hinder the nutritional intake of an infected individual. The skin lesions cause extensive perturbation of the skin, and this raises concerns about secondary bacterial infection of the skin, and this has been a complication that has been noticed in 19% of unvaccinated monkeypox patients [10,37]. The rash observed in infected individuals follows a very distinct presentation.

**Figure 7:**
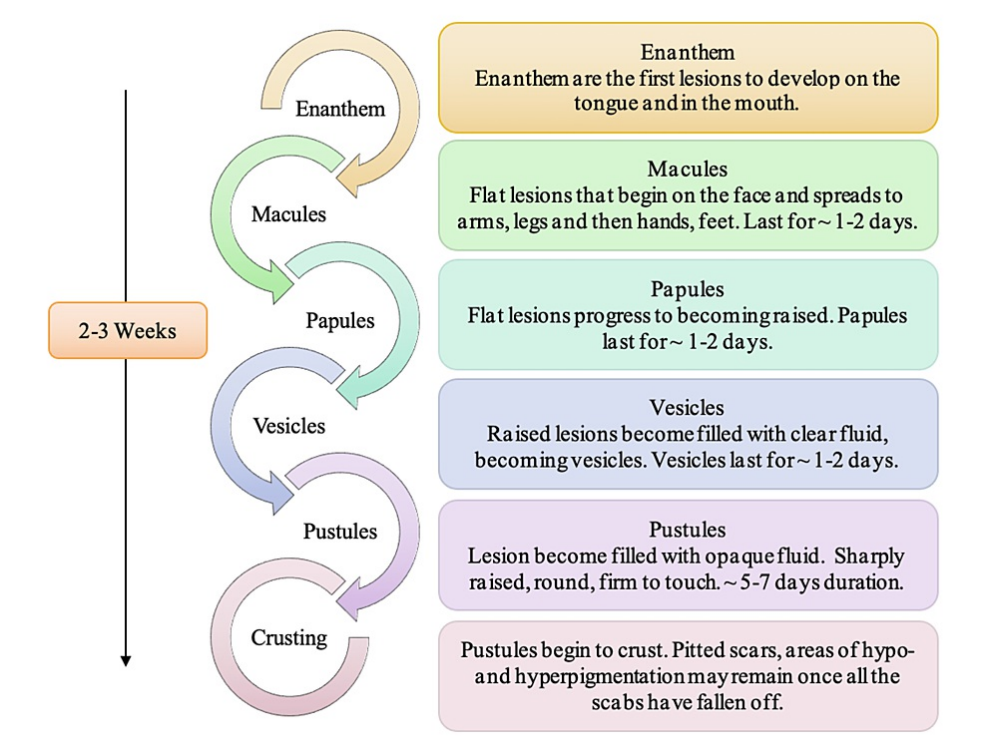
Stages of the Vesiculo-pustular Rash in Monkeypox Patients

The hallmark feature of monkeypox is a disseminated vesiculopustular rash [38]. The rash itself has been noted as going through several stages before they enter the desquamation phase in which the scabs begin peeling off. It has been noted that these distinctive lesions often present first as enanthem, macular, papular, then vesicular, and pustular, as is depicted in Figure 7 [10,39]. These lesions will ultimately become crusted within two to three weeks [40]. Prior to the rash appearing on the skin, an individual will see lesions developing on the tongue and mouth; these lesions are called enanthem. Once the crusted lesions peel off and reveal new skin underneath, an individual is no longer considered infectious. This is referred to as the desquamation phase. In some cases, individuals may be left with scars once scabs peel off. Some individuals may even present with regions of hyperpigmentation and hypopigmentation where the rash was more concentrated. The lesions are painful in all stages listed in Figure 7 until the desquamation phase, at which point, the crusting causes individuals extreme itchiness. Histopathologic analysis of the earliest stage of lesion development in humans reveals epidermal necrosis at the center of individual lesions concurrent with nascent extension into the superficial layers of the dermis [38,41]. It has also been observed in monkeypox-infected non-human primates that lesion pathology intensifies as pustules form, with progressive ulceration, necrosis, and interstitial hyperplasia [40]. Furthermore, edema is prominent at the margins of necrotic areas and clefts develop in interstitial spaces between cells where the fluid and cellular debris accumulates [20,38]. Eventually, inflammation and necrosis of the superficial dermis predominate, and destruction of sebaceous glands and follicles is evident [38]. Together, these attributes lead to the characterization of the affected areas as ‘partial-thickness wounds,’ and an injury of this extent points to the need for active prevention of complications such as secondary bacterial infections and possible cellulitis [38,41]. Interventional studies demonstrated that the use of moist occlusive therapies successfully promoted re-epithelialization and healing at herpes lesions sites and as such, the use of moist occlusive dressings could be contemplated for patients with extensive facial coverage of rash lesions [38,42].

Furthermore, gastrointestinal symptoms that arise by the second week of illness such as vomiting and diarrhea can contribute to severe dehydration in an infected individual [10]. The mouth and throat ulcers cause difficulties with maintaining nutrition, furthering chances of dehydration amongst patients. Of the other complications mentioned in Figure 6, the most serious complication of monkeypox is the corneal infection. Ocular infections may result in corneal scarring and permanent vision loss [10,43]. The majority of the complications associated with a monkeypox infection are detected in unvaccinated individuals (74%) than vaccinated patients (39.5%) [10]. Since the eradication of smallpox, the routine vaccine is no longer provided to today’s population. Due to cross-immunity, individuals that had been vaccinated against smallpox prior to the 1970s are much less likely to be developing the complications associated with a monkeypox virus infection. Sepsis and septic shock may also occur, and this is largely due to overly exaggerated immune responses [20,38]. Monkeypox is a self-limiting viral illness and due to the nature of the illness, lifelong complications are very rarely noted.

Although bronchopneumonia is a complication of monkeypox infection, it is a complication that is more commonly noted in individuals that are co-infected with the influenza virus [20]. The respiratory challenge of non-human primates across a range of infectious doses has been reproducibly shown to result in the development of focal necrosis of lung tissues, diffuse pulmonary consolidation, and fulminant bronchopneumonia [38]. Serious inflammation and bronchopneumonia can restrict air intake and diminish a patient’s willingness and/or ability to ingest food and fluids [10,38].

Clinical management and treatment

Currently, there are no specific treatments for monkeypox disease, however, experience with smallpox suggests that the vaccinia vaccine, cidofovir, tecovirimat, and vaccinia immune globulin (IVG) may have a use in monkeypox treatment [20]. As per the World Health Organization, tecovirimat was developed for smallpox and was licensed by the European Medicines Agency (EMA) for monkeypox in 2022 [9]. Currently, it is not yet widely available and as such, any use of tecovirimat should be monitored. Cidofovir has antiviral activity against a variety of viruses by inhibiting viral DNA polymerase [10]. Tecovirimat has been found to have specific efficacy on several orthopoxviruses, including variola, vaccinia, cowpox, ectromelia, rabbitpox, and monkeypox [20]. Tecovirimat is an oral intracellular viral release inhibitor with potential therapeutic effects on monkeypox [40].

Despite the suggested treatment interventions, supportive and symptomatic therapy has been deemed the basis of managing a monkeypox viral infection. Table 1 allows for insight into potential supportive treatment options that can be utilized to aid symptomatic individuals. It is important to understand that besides symptomatic management and preventing complications, there is no clear treatment for monkeypox. With the 2003 US outbreak of monkeypox and the current presentation of monkeypox cases, internationally, more research must be conducted before any treatment or vaccine can be commissioned.

**Table 1:** Symptoms/Complications and Potential Supportive Treatment

Symptom/Complication	Supportive Treatment
Respiratory distress/Bronchopneumonia	Oral/intravenous antibiotics for prophylaxis, nebulizer treatments, non-invasive ventilation (ex. CPAP)
Sepsis	Oral/intravenous antibiotics, supplemental oxygen, corticosteroids, insulin
Gastrointestinal/mouth and throat ulcers	Oral/intravenous antiemetic and antidiarrheal medications, oral/intravenous rehydration
Fever	Antipyretic medications, external cooling
Superinfection skin	Oral/intravenous antibiotics, incision, and drainage, advanced wound management (ex. negative pressure wound therapy
Inflammation/Lymphadenopathy	Oral/intravenous anti-inflammatory/analgesic medications
Corneal infection	Ophthalmic antibiotics/antivirals and corticosteroids
Skin scarring/Cellulitis/Skin lesions	Application of moist occlusive dressings to promote re-epithelization

## Conclusions

Previously endemic to regions of Africa, the monkeypox virus is now becoming a global concern, with sporadic cases being confirmed in regions in the western hemisphere. With human-to-human transmission most commonly occurring via respiratory droplets or direct contact with the mucocutaneous lesions of an infected individual, social distancing and contact tracing is imperative. Monkeypox cases are being confirmed in mid-age individuals. This can be attributed to the loss of cross-immunity from the smallpox vaccine seen in older individuals. This virus replicates within the cytoplasm and matures to create a primary viremia in which the virus spreads to the local lymph nodes. Monkeypox infection is also associated with complications such as bronchopneumonia, dehydration, respiratory distress, encephalitis, etc. Of all the complications, the most feared complication is corneal scarring, as it can lead to vision loss. It is important to be able to provide the appropriate supportive treatment to ensure that the risk of these complications can be minimized as much as possible. Supportive therapy, such as applying moist occlusive dressings, may be applied in areas where the rash is highly concentrated. As cases of monkeypox cases are still being confirmed globally, organizations are focused on understanding how these cases are sporadically occurring across Europe and the western hemisphere. Investigation into any potential treatments is important, along with understanding the true extent of all the symptoms of monkeypox and the long-term effects of the virus and the symptoms.
